# Plasma COL3A1 propeptide as a promising prognostic marker in oral squamous cell carcinoma

**DOI:** 10.1038/s41598-026-57329-0

**Published:** 2026-06-29

**Authors:** Mustafa Magan, Xiaolian Gu, Nicola Sgaramella, Karin Nylander

**Affiliations:** 1 Department of Medical Biosciences, Umeå University, Building 6M, 901 85 Umeå, Sweden; 2 Department of Clinical Sciences/ENT, Umeå University, 901 87 Umeå, Sweden; 3 Department of Oral and Maxillo-Facial Surgery, Mater Dei Hospital, 70125 Bari, Italy

**Keywords:** Oral squamous cell carcinoma, Collagen, COL3A1, Tumour microenvironment, Biomarker, Extracellular matrix, Biomarkers, Cancer, Oncology

## Abstract

In this study, we aimed to evaluate the prognostic significance of collagens in patients with oral squamous cell carcinoma (OSCC). Plasma proteomics data from 40 OSCC patients and 33 healthy controls, generated using the Olink Explore 3072 platform, along with microarray gene expression data from 29 OSCC tumours, 21 clinically normal tissues contralateral to tumour (NTCT), and 14 healthy controls, were analysed to assess expression levels of collagens and their associations with clinicopathological variables and survival outcomes. Eleven collagen peptides were detected in plasma. Higher circulating levels of collagen type III alpha 1 chain (COL3A1) propeptides were associated with improved overall survival and longer disease-free time (p < 0.05). Penalised Cox regression using the least absolute shrinkage and selection operator (LASSO) supported COL3A1 propeptide as a survival-associated feature. Comparison of protein profiles between COL3A1-low and COL3A1-high patients identified neutrophil degranulation as the most significantly enriched pathway. Microarray differential expression analysis showed COL3A1 mRNA to be significantly upregulated in tumour tissues compared with NTCT and healthy controls (FDR < 0.05). Circulating COL3A1 propeptide shows potential as a prognostic biomarker for OSCC and may reflect systemic immune-related alterations.

**Supplementary Information:**

The online version contains supplementary material available at 10.1038/s41598-026-57329-0.

## Introduction

Head and neck squamous cell carcinoma (HNSCC) is a complex malignant disease accounting for approximately 90% of cancers in the head and neck region. It is also the sixth most prevalent malignancy worldwide^1,2^ The incidence is increasing globally, with approximately 890,000 new cases and 450,000 deaths annually^1^. Oral cavity squamous cell carcinoma (OSCC) is one of the most common subtypes of all HNSCC^3^, and in developing countries OSCC accounts for approximately two-thirds of the HNSCC cases^4^.

Primary treatment of these tumours includes surgery, radiotherapy, or a combination of both. Although additional modalities such as cisplatin-based chemotherapy and targeted molecular therapies have been introduced, the five-year overall survival rate is still low, ranging from 55 to 66%^5,6^. Within two years after treatment, about 30–50% of patients with locoregional advanced HNSCC experience a recurrence^7^. So far, there are no clinically useful markers for early tumour detection or post-therapeutic monitoring of the disease^5^.

The tumour microenvironment (TME) in HNSCC comprises immune cells, stromal cells, extracellular matrix elements (ECM), and secreted factors that influence tumour progression, metastasis, and treatment response^8^. Within the TME, cancer-associated fibroblasts (CAFs) are the most prominent cell type, and the ECM the most abundant non-cellular network^9^. CAFs can originate from diverse sources in HNSCC but are primarily derived through differentiation of local normal fibroblasts, through TGF-β1 and IL-1β signalling from tumour cells^10^.

The crosstalk between CAFs and cancer cells critically influences the progression of OSCC through multiple mechanisms, including proliferation, invasion, metastasis and evasion of immune cell surveillance^11^. CAFs mediate the secretion of various factors known to promote tumour proliferation, invasion and fibrosis, such as IGF-2 and TGF-β, thereby creating a favourable tumour microenvironment^4,12,13^. The ECM, which primarily comprises collagens derived from CAFs, undergoes substantial remodelling, which in turn facilitates tumour migration and invasion^14–16^. To date, at least 28 distinct collagen types have been identified, each with a unique molecular composition, tissue distribution, and biological function. In tumours, dysregulation of collagens type I, III, IV, V, and VI has been widely reported^15^.

During collagen formation, N- and C-terminal propeptides are cleaved from pro-collagen molecules and released into the circulation, where they can be detected in peripheral blood as indicators of collagen synthesis. In contrast, protease-mediated degradation of mature collagen generates soluble collagen fragments that subsequently enter the bloodstream^17^. Measurement of collagen-derived peptides, including propeptides and degradation fragmentsin blood, therefore, offers a minimally invasive approach to assess tumour-associated tissue remodelling, as well as systemic ECM remodelling^18^. In several malignancies, elevated circulating levels of collagen-derived peptides have been linked to aggressive disease and poorer clinical outcomes^19–21^. This study aimed to assess the clinical relevance of circulating collagen-derived peptides in patients with OSCC. As a measure of local ECM remodelling, collagen mRNA expression in OSCC tumour tissue was also analysed.

## Materials and methods

### Patient material and data

Data from the proteomics and microarray analyses have been previously published^22,23^. The studies were approved by the Regional Ethical Review Board in Umeå, Sweden (Dnr 03–201; 08 − 003 M; 2012-131-33 M and 2014-193-32 M). Written informed consent was obtained from all participants.

In the proteomics study, blood samples were collected from 40 patients and 33 healthy controls, with a mean age of 56.5 and 58 years, respectively. Three millilitres of peripheral blood was obtained via venipuncture into heparin-treated collection tubes and left at room temperature for 30 min. Thereafter, they were centrifuged at 1300 g for 10 min at room temperature, and the supernatant, primarily consisting of plasma, was decanted into separate vials and stored at − 80 °C until further use. All samples were collected consecutively between November 2012 and October 2021 at the Neuro, Head and Neck Centre at Norrland´s University Hospital (Umeå, Sweden). Patient characteristics are shown in Table S1. Proteomic profiling was conducted using the Olink Explore 3072 platform (Olink Proteomics, Uppsala, Sweden)^23^. A total of 13 collagen assays are included on this platform. Antibodies for COL1A1, COL3A1 and COL5A1 detect propeptides (representing collagen synthesis) while antibodies for the other 10 collagen types detect mature collagen epitopes (representing degradation fragments) (Table S2).

The microarray study included 31 patients with squamous cell carcinoma of the oral tongue (SCCOT) and 14 healthy controls (HC). Tumour biopsies (*n* = 29) and/or clinically normal tissue contralateral to the tumour (NTCT, *n* = 21) were collected before treatment. Healthy individuals provided biopsies from the lateral border of the tongue (*n* = 14). The mean age was 61 for tumour patients and 40 years for HC. Patient characteristics are presented in Table S3. All tissue samples were immediately frozen in liquid nitrogen and stored at −80 °C until RNA extraction. As described previously, gene expression profiling was performed using Illumina HumanHT-12 v4 Expression BeadChip (Illumina Inc., San Diego, CA, USA). Normalisation of microarray data was performed using linear models and the differential expression for microarray data (LIMMA) package. Raw data were deposited in ArrayExpress (accession numbers E-MTAB-4678 and E-MTAB-5534)^22^.

### Statistical analysis

The Wilcoxon rank-sum test with false discovery rate (FDR) adjustment was used to evaluate differences in plasma protein levels between patients and healthy controls. Overall survival (OS) defined as time from completion of initial treatment until death from any cause, and disease-free survival (DFS) defined as time from completion of initial treatment until either first documented disease recurrence or death without evidence of recurrence, were analysed using Kaplan–Meier survival analysis with log-rank tests. Patients were stratified into high and low abundance groups based on the median value.

To identify potentially independent prognostic factors, multivariate Cox proportional hazards regression models were applied. To reduce overfitting given the limited number of events relative to the number of covariates, penalised Cox proportional hazards regression with the least absolute shrinkage and selection operator (LASSO) was performed in R using the glmnet package. Candidate variables included the protein of interest, age, sex, tumour size, and nodal status. The optimal penalty parameter (lambda) was determined by 10-fold cross-validation based on the minimum mean cross-validated error (lambda.min). Variables with non-zero coefficients at lambda.min were considered retained predictors.

To compare mRNA expression levels between tumour and non-tumour samples ComparativeMarkerSelection analysis was performed using the GenePattern platform (v10, https://www.genepattern.org). An FDR < 0.05 or a two-sided *p*-value < 0.05 was considered statistically significant. Statistical analyses were performed using SPSS (version 29, IBM Corp., Armonk, NY, USA) or R (version 4.5.2; R Foundation for Statistical Computing, Vienna, Austria). Boxplots were generated using the ggplot2 package, and Kaplan-Meier survival curves were plotted using the survival and survminer packages.

### Functional enrichment analysis

To assess the functional implications of survival-associated proteins, differential abundance analysis was performed to compare protein profiles between high- and low-abundance groups defined by each survival-associated protein. Differential protein expression was analysed using a linear modelling approach within a generalised linear model (Gaussian distribution) framework implemented in the *limma* package in R. Protein abundance was modelled as a function of protein-defined group with adjustment for age as a covariate. Empirical Bayes moderation was applied to improve variance estimation, and *p*-values were corrected for multiple testing using the Benjamini-Hochberg method. Proteins showing significant differences between the two groups (*p* < 0.05) were then subjected to downstream exploratory functional enrichment analysis using Enrichr with a focus on Reactome pathways^24–26^.

## Results

### Plasma protein levels

**Fig. 1:**
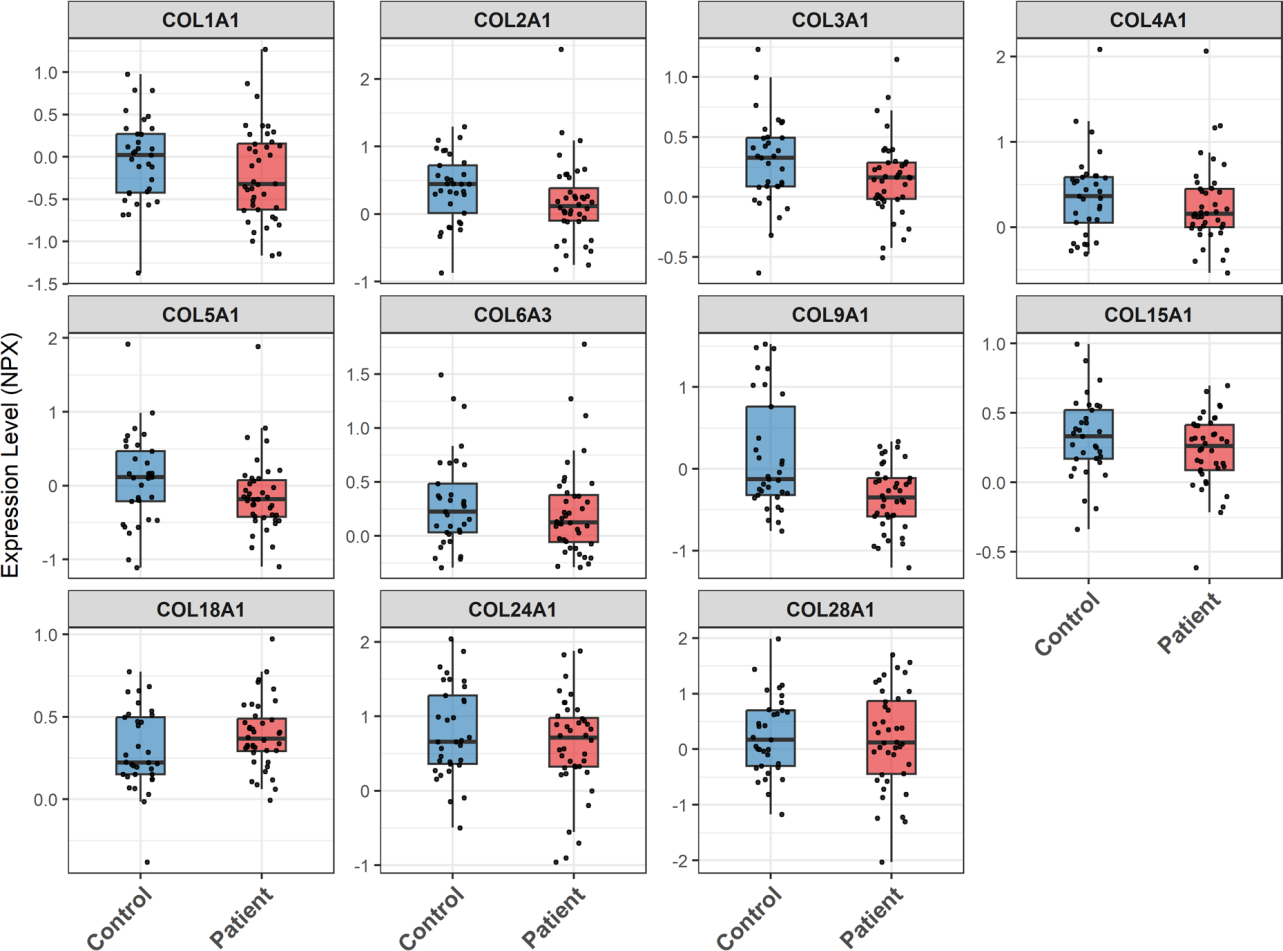
Plasma levels of collagen turnover markers in patients and controls are shown as box plots of normalised protein expression (NPX) levels.

Among the proteins measured on the Olink Explore 3072 platform, 11 collagen-derived peptides were detected in plasma from patients and controls (COL1A1, COL2A1, COL3A1, COL4A1, COL5A1, COL6A3, COL9A1, COL15A1, COL18A1, COL24A1, COL28A1). When comparing plasma protein levels between patients and controls, no significant differences were seen (Fig. 1).

### Comparison of plasma collagen levels with clinical parameters

We next investigated whether collagen-derived peptide levels vary with age, sex, tumour size, and lymph node status. Seven peptides (COL1A1, COL2A1, COL3A1, COL5A1, COL6A3, COL9A1, and COL18A1) were significantly associated with age (*p* < 0.05), with the highest correlation seen for COL18A1 (Spearman’s *rho* = 0.538). When comparing early-stage (T1 + T2) and advanced-stage disease (T3 + T4) of OSCC, COL24A1 levels were significantly higher in T1 + T2 than in T3 + T4 tumours (*p* = 0.002). No significant differences in collagen peptide levels were observed based on sex or cervical lymph node status (Table S4).

### Impact of plasma collagen protein levels on survival outcome

**Fig. 2:**
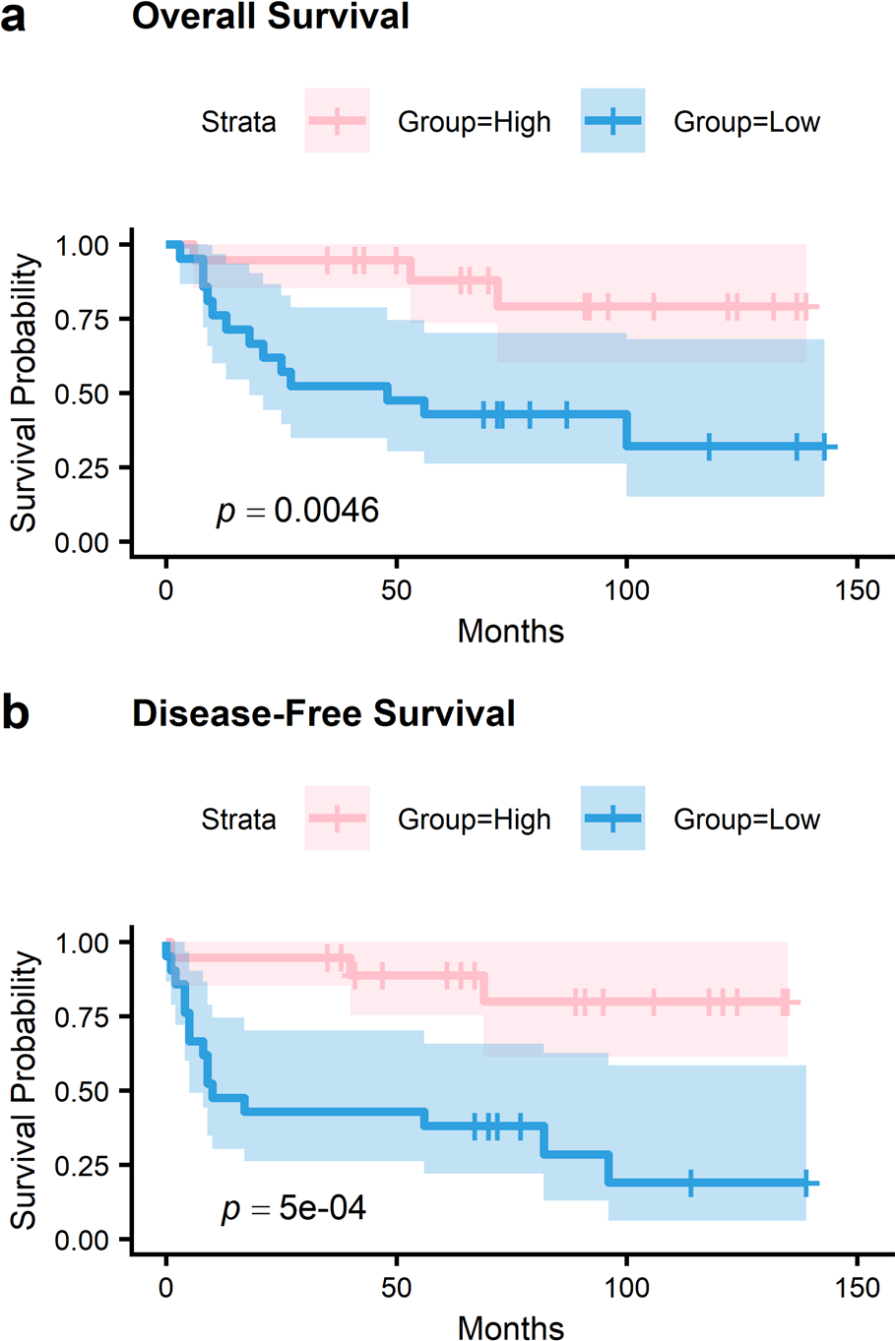
Kaplan-Meier plots showing the association of plasma COL3A1 levels with survival. (**a**) Overall survival; (**b**) disease-free survival. Patients were stratified into high- and low-groups based on the median COL3A1 level. Survival differences were assessed using the log-rank test.

To investigate whether plasma peptide levels influenced the overall survival (OS) and disease-free survival (DFS), OSCC patients were stratified into high and low groups based on the median protein level, and Kaplan-Meier survival analysis with the log-rank test was performed. Patients with high plasma levels of COL3A1 propeptides demonstrated significantly longer OS than those with low protein levels over a 12-year follow-up time (*p* = 0.0046; Fig. 2a). Similarly, high COL3A1 propeptide levels were associated with significantly longer DFS (*p* = 0.00054; Fig. 2b). Variations in plasma levels of the other 10 collagen peptides did not significantly affect OS or DFS (data not shown).

Following the observation that elevated plasma levels of COL3A1 propeptides were associated with improved survival, multivariate analyses were conducted to evaluate their independent prognostic significance alongside established clinicopathological parameters (age, sex, tumour size, and nodal status). As shown in Table 1, Cox proportional hazards regression analysis demonstrated that high plasma levels of COL3A1 propeptides were independently associated with better OS (*p* = 0.007, hazard ratio = 0.169, 95% confidence interval = 0.046–0.618) and DFS (*p* < 0.001, hazard ratio = 0.105, 95% confidence interval = 0.028–0.399).

**Table 1:** Multivariate Cox proportional hazards regression analysis.

Variable	Overall survival			Disease-free survival		
	*P*-value	Hazard ratio	95% CI	*P*-value	Hazard ratio	95% CI
COL3A1 (high vs. low)	0.007	0.169	0.046–0.618	< 0.001	0.105	0.028–0.399
Age at diagnosis	0.234	1.022	0.986–1.060	0.513	1.011	0.979–1.044
Sex	0.586	0.623	0.114–3.414	0.94	0.943	0.204–4.348
Tumour size	0.428	1.556	0.522–4.635	0.169	2.108	0.729–6.095
Nodal status	0.031	5.75	0.522–4.635	0.044	4.225	1.041–17.141

LASSO-penalised Cox regression with COL3A1, age, sex, tumour size, and nodal status as candidate variables retained COL3A1, tumour size, and nodal status in the models for both OS and DFS (Table 2). For OS, higher COL3A1 level was associated with a reduced risk of death (hazard ratio = 0.33), whereas larger tumour size (hazard ratio = 1.39) and positive nodal status (hazard ratio = 1.31) were associated with increased risk. For DFS, higher COL3A1 level was similarly associated with a reduced risk of recurrence (hazard ratio = 0.23), while larger tumour size (hazard ratio = 1.40) and positive nodal status (hazard ratio = 1.38) were associated with increased risk.

**Table 2:** LASSO-Penalised Cox Results.

	Overall survival			Disease-free survival		
	Coefficient	Hazard ratio (exp(coef))	Retained by LASSO	Coefficient	Hazard ratio (exp(coef))	Retained by LASSO
COL3A1	−1.11	0.33	Yes	−1.46	0.23	Yes
Tumour size	0.33	1.39	Yes	0.34	1.4	Yes
Nodal status	0.27	1.31	Yes	0.32	1.38	Yes
Age	0	1	No	0.01	1.01	No
Sex	0	1	No	0	1	No

### Plasma proteomic differences between COL3A1-low and COL3A1-high patients

**Fig. 3:**
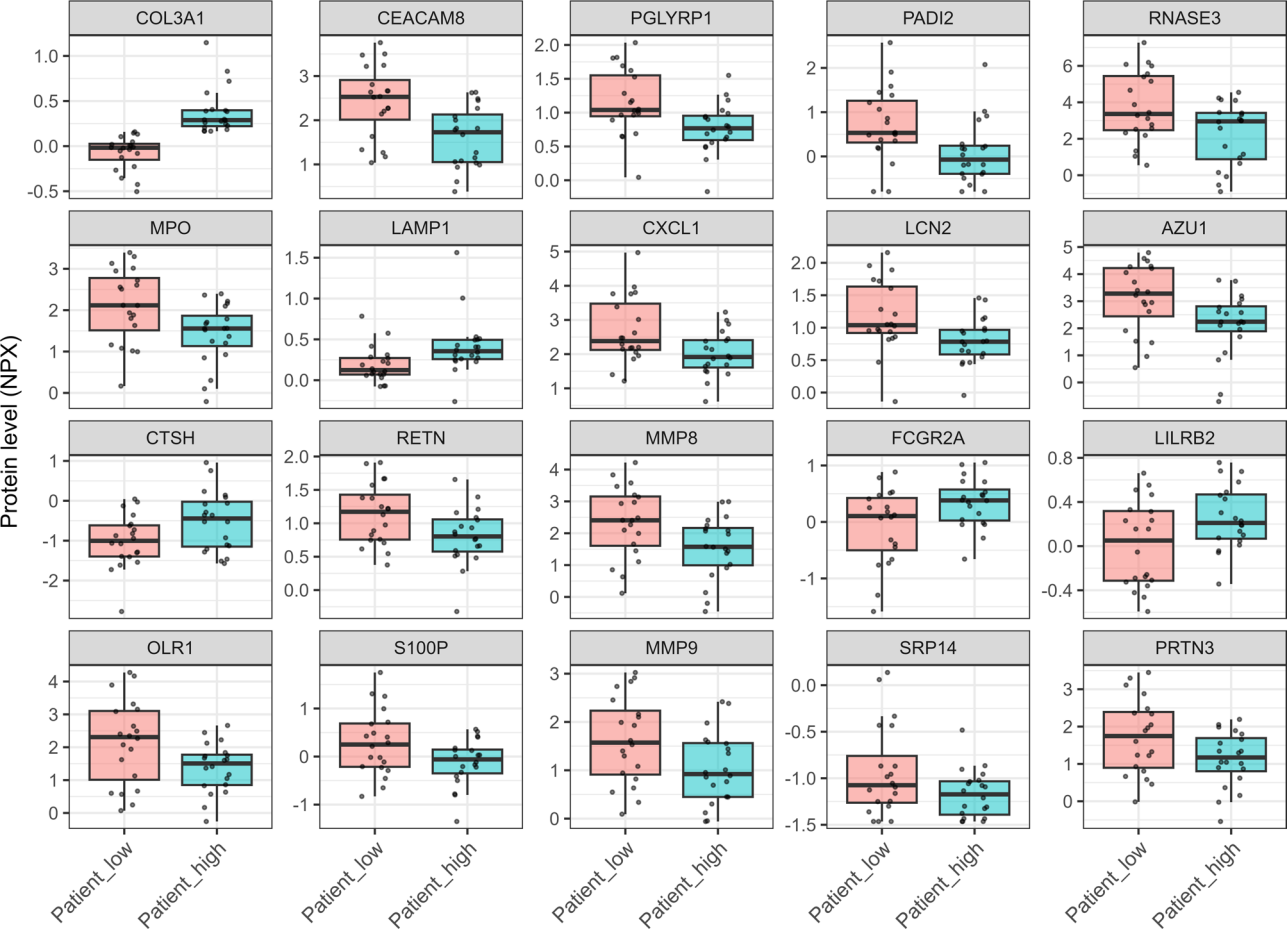
Box plots of plasma levels of the 19 proteins involved in the neutrophil degranulation pathway, comparing patients with high COL3A1 expression (patient_high) to those with low COL3A1 expression (patient_low).

To assess the functional relevance of COL3A1 levels in relation to survival, plasma proteomic profiles were compared between COL3A1-low and COL3A1-high patients. Age-adjusted generalised linear modelling identified 112 proteins with *p* < 0.05, however, only COL3A1 remained significant after multiple testing correction (adjusted *p* < 0.05, Table S5). For exploratory analysis, these 112 proteins were subjected to Reactome pathway enrichment analysis, which identified neutrophil degranulation as the most significantly enriched pathway (Table 3). A total of 19 proteins contributed to this enrichment, including key neutrophil granule proteins such as CEACAM8, PGLYRP1, and RNASE3. Notably, elevated plasma levels of these proteins were observed in COL3A1-low patients (Fig. 3).

**Table 3:** The top three significantly enriched Reactome pathways.

Term	Overlap	*P*-value	Adjusted *P*-value	Proteins
Neutrophil Degranulation	19/478	1.96E-11	1.26E-08	CXCL1;RNASE3;AZU1;RETN; LILRB2;SRP14;MMP8;MPO; MMP9;FCGR2A; LAMP1;LCN2;PADI2;CTSH; OLR1;PRTN3;S100P; CEACAM8;PGLYRP1
Immune System	32/2150	1.50E-07	4.83E-05	LILRA6;CXCL1;RETN; SRP14;MPO; LAMP1;TPR; EP300;CTSH; OLR1;PGLYRP1;JUN; OSM; FLT3LG; RNASE3;AZU1;LILRB2;MMP8;MMP9;MAPK13;COL1A1;COL3A1;FCGR2A; PRKRA; CD207;LCN2;CDK1;PADI2;PRTN3;S100P; CEACAM8;VIM
Innate Immune System	22/1149	3.42E-07	7.33E-05	JUN; CXCL1;RNASE3;AZU1;RETN; LILRB2;SRP14;MMP8;MPO; MMP9;MAPK13;FCGR2A; LAMP1;LCN2;EP300;PADI2;CTSH; OLR1;PRTN3;S100P; CEACAM8;PGLYRP1

### Tissue mRNA levels

**Fig. 4:**
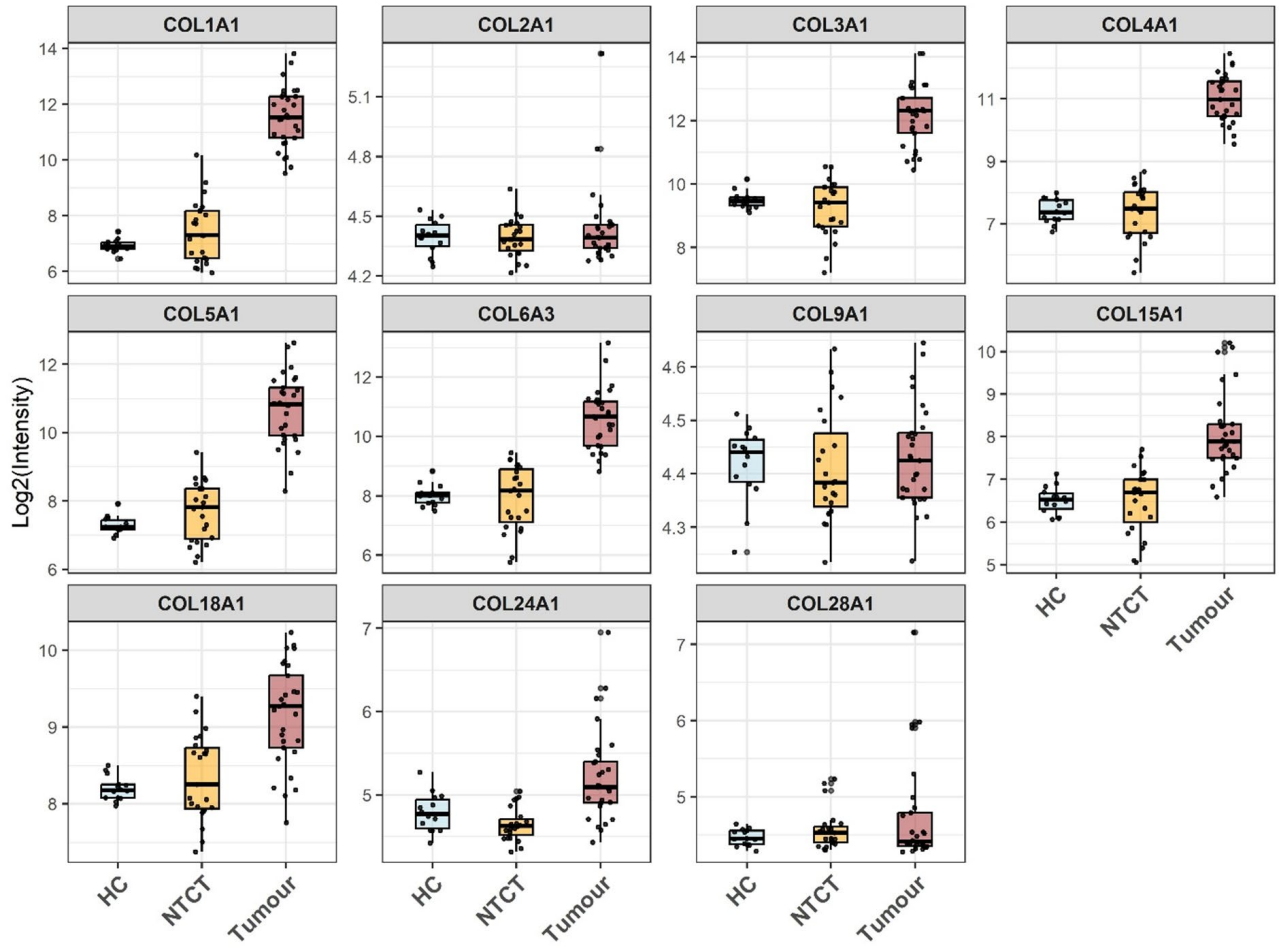
Box-plots showing collagen mRNA levels in healthy controls (HC), clinically normal tongue tissue contralateral to tumour (NTCT) and tumour samples.

To clarify status of the 11 collagens in local ECM remodelling, microarray gene expression data were analysed to assess mRNA levels in 29 OSCC samples from the oral tongue, 21 clinically normal tissue contralateral to the tumour (NTCT), and 14 healthy controls (HC). Differential gene expression analysis revealed significantly higher mRNA levels of 8 genes (COL1A1, COL3A1, COL4A1, COL5A1, COL6A3, COL15A1, COL18A1, COL24A1) in tumour samples compared to NTCT and HC (FDR < 0.05). No significant differences were seen between NTCT and HC biopsies (Fig. 4).

### Comparison of tumour collagen levels with clinical parameters

Since most of the analysed genes showed higher mRNA levels in tumour samples, we next investigated whether these expression differences were associated with age, sex, tumour size, or nodal status. No significant correlations were, however, observed with any of these parameters (data not shown).

### Impact of collagen mRNA levels on survival outcome

**Fig. 5:**
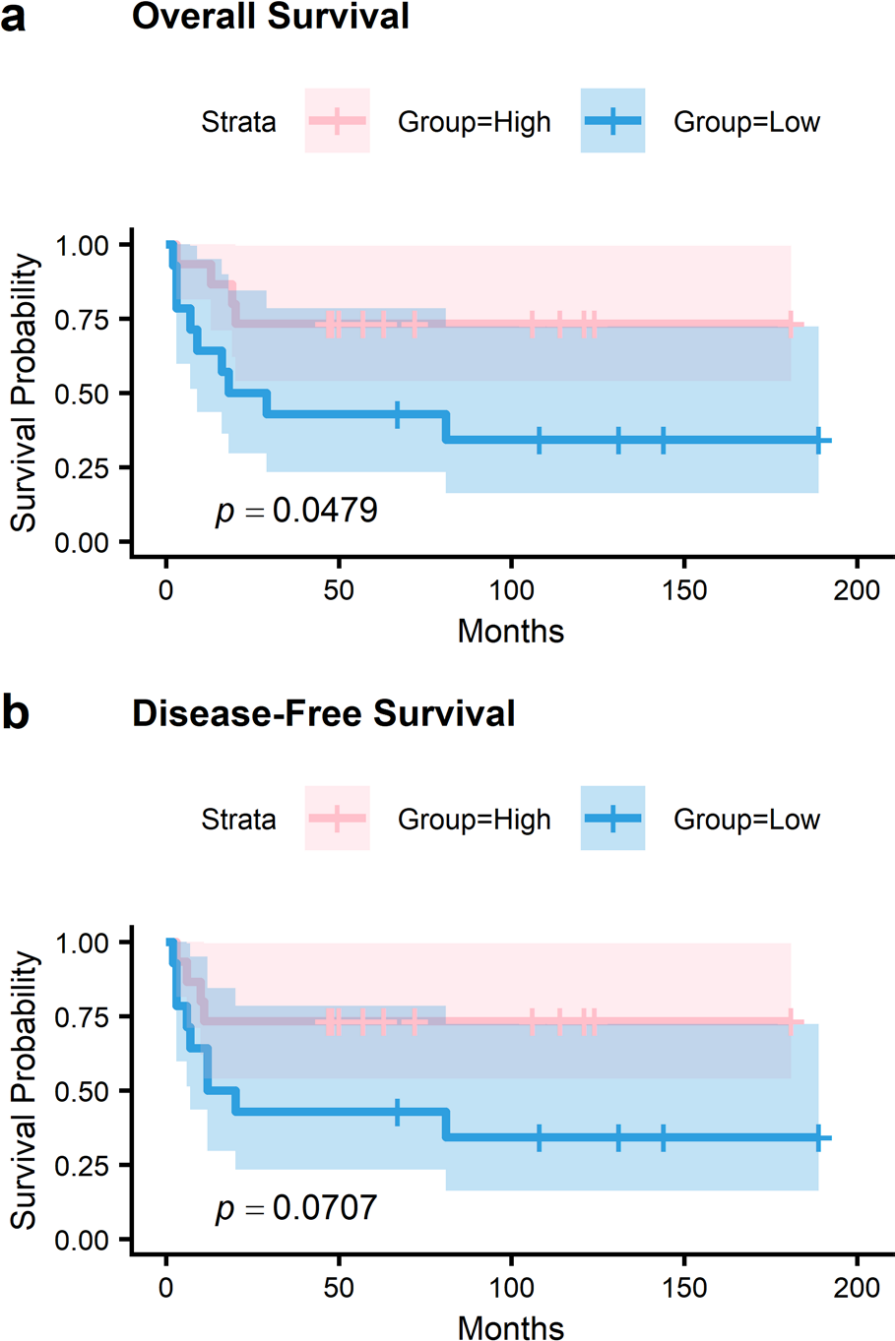
Kaplan-Meier plots showing the association of tumour *COL3A1* levels with survival. (**a**) Overall survival; (**b**) disease-free survival. Patients were stratified into high- and low-groups based on the median *COL3A1* level. Survival differences were assessed using the log-rank test.

The mRNA levels of the collagen genes were further analysed for their potential association with OS and DFS. Kaplan-Meier log-rank analysis showed that only *COL3A1* was significantly correlated with OS (*p* = 0.048; Fig. 5a) and also showed a non-significant trend toward an impact on DFS (*p* = 0.071; Fig. 5b). After adjusting for age, sex, tumour size, and nodal status, Cox proportional hazards regression analysis, however, showed that tumour *COL3A1* mRNA levels were not independently associated with overall survival.

## Discussion

The current study focused on the most abundant non-cellular component in the TME, collagens, to better understand their role in predicting patients’ prognosis. By analysing plasma samples and tumour biopsies from OSCC patients, we investigated whether circulating collagen-derived peptides and their corresponding genes exhibit differential expression and possess prognostic value.

Our proteomic analysis identified 11 collagen-derived peptides in plasma, with similar levels observed in patients and healthy controls. At the mRNA level, eight corresponding collagen genes were upregulated in tumour tissue compared with both contralateral normal samples and healthy controls. Although the microarray cohort exhibited a substantial age difference between healthy controls (mean 40 years) and tumour patients (mean 61 years), the observed upregulation of collagen mRNA appears to be driven by tumour status rather than age, as clinically normal contralateral tissue (NTCT) did not differ significantly from healthy controls, whereas matched tumour versus NTCT comparisons within the same individuals showed clear differences.

Apart from significant correlations with age for several collagen peptides and significantly higher levels of COL24A1 peptides seen in smaller tumours (T1 + T2), the analysed collagens showed no correlations with sex or nodal status, either at the circulating peptide or local mRNA level. In survival analyses, higher plasma levels of COL3A1 propeptides were independently associated with better OS as well as DFS. Notably, *COL3A1* mRNA was elevated in tumour tissues but was not independently associated with either OS or DFS.

COL3A1 is a fibrillar-forming collagen in the interstitial matrix and the second most abundant collagen in the body as well as a crucial component of the ECM. In peripheral blood, several studies of different solid tumours have demonstrated elevated levels of COL3A1 propeptides to be associated with poor prognosis^19,27–29^. In the measurement of COL3A1 in blood, different methods can be used. We applied measurement of the C- and N-terminus propeptides, which are released under active collagen formation^30^. An alternative is to measure so-called N-terminal telopeptides which are released during collagen degradation, as previously demonstrated by Nurmenniemi et al.^31^. In their study, by looking at the N-terminal telopeptide of COL3A1 in sera from patients with HNSCC high levels showed a connection to poor prognosis, even though not statistically significant. This result is in accordance with the present study where an association between high levels of C-terminal propeptides released during synthesis of COL3A1 and better OS and DFS was seen, whereas they saw an association between high levels of a marker for degradation of COL3A1 and poor prognosis^31^.

Comparing patients to controls, tumour tissue *COL3A1* mRNA expression was elevated whereas circulating COL3A1 propeptides remained similar. Notably, the plasma cohort comprises multiple oral cavity subsites whereas the microarray tissue cohort is restricted to the oral tongue. Therefore, the tissue analyses provide complementary evidence of local ECM remodelling rather than a direct validation of the plasma results. Nevertheless, this discrepancy likely reflects the predominantly local nature of collagen deposition within the tumour microenvironment, as well as the fact that circulating collagen-derived peptides reflect overall extracellular matrix turnover rather than tumour-specific expression alone. In addition to tumour-associated remodelling, these markers may be influenced by non-malignant fibrotic or inflammatory processes in other tissues, while the presence of such conditions in the healthy controls was unknown. As procollagen propeptides are released into the circulation during collagen synthesis, contributions from multiple tissues may dilute tumour-derived signals in plasma^32–34^. Collectively, these observations suggest that plasma COL3A1 propeptides may have greater utility for prognostic stratification than for diagnostic discrimination in OSCC. Across multiple analytical approaches, higher plasma COL3A1 levels consistently emerged as a favourable prognostic factor. Univariate Kaplan-Meier analysis demonstrated improved survival for patients with high COL3A1, traditional multivariable Cox regression showed a protective effect after adjusting for clinical covariates, and LASSO-penalised Cox regression supported COL3A1 as a key predictor rather than a statistical artefact.

In OSCC, COL3A1 may, on the one hand, promote the formation of a dense extracellular matrix that physically restricts tumour cell migration and potentially contributes to improved patient survival. On the other hand, this matrix barrier could hinder the delivery of therapeutic agents and restrict immune infiltration at the tumour site. When co-transplanting CAFs, the main producers of COL3A1, with HNSCC cells into the mouse oral mucosa, distinct CAF behaviours were seen. CAF-P (promotor) surrounded cancer cells and promoted cancer cell invasion, whereas CAF-D (defence) formed a demarcated boundary and did not induce invasiveness. Interestingly, CAF-D showed increased COL3A1 expression levels^35^, indicating that higher COL3A1 levels could exert antitumor effects. Therefore, patients with high COL3A1 levels in tumour tissues may exhibit a paradoxical phenotype characterised by a more favourable baseline prognosis but potentially poorer responsiveness to certain systemic and immunological therapeutic approaches. This complexity is consistent with our finding that *COL3A1* level in tumour tissue was not an independent predictor of survival.

In comparing plasma proteomic profiles between COL3A1-low and COL3A1-high patients, exploratory enrichment analysis of nominally differentially abundant proteins identified neutrophil degranulation as the most enriched pathway. This pathway included key neutrophil granule proteins, many of which were elevated in COL3A1-low patients. In line with prior evidence that tumour-associated neutrophils in OSCC contribute to extracellular matrix degradation, tumour invasiveness, and adverse clinical outcomes^36^, our findings raise the possibility that low circulating COL3A1 reflects a neutrophil-associated inflammatory state. Although direct evidence for neutrophil degranulation in HNSCC remains limited, accumulating data in OSCC/HNSCC support the involvement of neutrophil-related inflammatory programs in tumour progression, including associations with invasion, metastasis, and poor prognosis^37–39^. Proteomic evidence from early-stage gallbladder cancer further supports this biological link, as proteins related to neutrophil degranulation were identified alongside those involved in extracellular matrix remodelling^40^. Taken together, these findings suggest that elevated circulating COL3A1 levels are associated with a less inflammatory systemic profile, which mayreflect a more contained tumour microenvironment, whereas low COL3A1 levels may reflect a more neutrophil-driven and potentially more aggressive phenotype. This may partly account for the improved survival observed in patients with high COL3A1 levels. Notably, extracellular matrix remodelling in the tumour microenvironment is driven by multiple immune cell populations. Tumour-associated macrophages (TAMs), in particular, are recognised contributors to collagen turnover and matrix degradation through the secretion of matrix-remodelling enzymes and pro-fibrotic mediators^41^. The enrichment of immune system and innate immune system pathways further supports the involvement of a broader immune-stromal interaction network, particularly in the context of systemic inflammatory responses.

In summary, plasma COL3A1 propeptides may serve as a prognostic biomarker in OSCC. Of note, age was inversely correlated with plasma COL3A1 propeptide levels and is also an established determinant of overall survival, highlighting its potential role as a confounding factor. Although age was included as a covariate in the multivariable Cox model and COL3A1 remained independently associated with survival outcomes, the limited number of events restricts the robustness of this adjustment. Therefore, residual confounding cannot be fully excluded by statistical modelling alone. Consequently, the robustness and clinical applicability of these findings require validation in an independent cohort with an adequate number of events. Furthermore, larger paired tumour-plasma datasets will be necessary to clarify the relationship between tumour-derived ECM components and their representation in circulation. In addition, the lack of a significant difference in plasma COL3A1 levels between patients and healthy controls limits the interpretation of what constitutes a “high” value in a clinical setting. The establishment of clinically meaningful and absolute thresholds will be required.

## Supplementary Information

Below is the link to the electronic supplementary material.

Supplementary Material 1

## Data Availability

Microarray raw data were deposited in ArrayExpress under accession numbers E-MTAB-4678 and E-MTAB-5534. Plasma proteomics data are available from the corresponding author upon reasonable request.
