# Supplementary material for: Plasma COL3A1 propeptide as a promising prognostic marker in oral squamous cell carcinoma
Source: Sci Rep. 2026 Jun 29;16:19773. doi: 10.1038/s41598-026-57329-0 (PMC13314942; doi:10.1038/s41598-026-57329-0)
Supplement: Supplementary file 1 — Supplementary Material 1 [file 41598_2026_57329_MOESM1_ESM.pdf]

Plasma COL3A1 Propeptide as a Promising Prognostic Marker in Oral Squamous Cell Carcinoma

Mustafa Magan[1,2], Xiaolian Gu[1], Nicola Sgaramella[1,4], Karin Nylander[1]

Mustafa Magan[1,2], Xiaolian Gu[1], Nicola Sgaramella[1,3], Karin Nylander[1]

[1]Department of Medical Biosciences, Building 6M, Umeå University, 901 85 Umeå, Sweden; [2]Department of Clinical Sciences/ENT, Umeå University, Umeå, Västerbotten, 901 87, Sweden; [3]Department of Oral and Maxillo-Facial Surgery, Mater Dei Hospital, 701 25, Bari, Italy

Table S1. Clinicopathological parameters of the enrolled patients with OSCC.

ID	Sex	Age	Tumor site	TNM	Therapy	Follow-up (months)	Status	Recurrence
98	M	31	Tongue	cT2N0M0	S+Postop RT	143	Alive	No
101	M	69	Gingiva	pT4aN1M0	Preop RT+S	139	Alive	No
104	F	69	Gingiva	cT4aN0M0	S+ Postop RT	137	Alive	No
105	M	63	Tongue	cT1N0M0	Preop RT+S	137	Alive	Yes
111	F	31	Tongue	cT1N0M0	S+Postop RT	132	Alive	No
120	M	29	Buccal	cT2N2bM0	Preop RT+S	56	Dead	No
127	M	27	Tongue	pT1N1M0	S+Postop RT	124	Alive	No
129	M	59	Gingiva	cT3N0M0	S+Postop RT	122	Alive	No
131	F	74	Tongue	pT2N0M0	S+Postop RT	100	Dead	No
133	M	33	Gingiva	cT4bN2bM0	S+Postop RT	27	Dead	No
137	F	71	Tongue	cT2N0M0	Preop RT+S	118	Alive	No
138	M	37	Tongue	cT2N1M0	Preop RT+S	72	Dead	No
143	F	37	Gingiva	pT4aN0M0	S+Postop RT	10	Dead	No
155	F	84	Tongue	cT2N0M0	S	18	Dead	No
157	M	68	Tongue	pT1N0M0	S	106	Alive	No
162	F	78	Gingiva	cT1N0M0	S	9	Dead	No
187	F	73	Tongue	pT1N0M0	S	96	Alive	No
189	M	73	Tongue	pT4aN1M0	S+Postop RT	8	Dead	Yes
199	M	56	Gingiva	cT4N0M0	S+Postop RT	92	Alive	No
200	F	66	Buccal	cT1N0M0	S	91	Alive	No
202	F	78	FOM	pT2N2bM0	S+Postop RT	6	Dead	Yes
212	M	52	Tongue	T4aN2bM0	RT+CT	13	Dead	Yes
213	F	72	Tongue	pT3N0M0	S+Postop RT	87	Alive	No
225	M	55	Buccal	cT4aN1M0	RT+CT	8	Dead	No
226	M	62	FOM	pT1N0M0	S	79	Alive	No
231	M	62	Tongue	pT2N2bM0	S+Postop RT	73	Alive	No
242	F	54	Tongue	cT1N0M0	S	72	Alive	No
246	M	36	Tongue	cT2N0M0	S+Postop RT	70	Alive	No
248	M	39	Tongue	cT2N0M0	S+Postop RT	69	Alive	No
253	M	69	Tongue	cT2N0M0	S+Postop RT	53	Dead	No
268	F	52	Tongue	pT1N0M0	S	66	Alive	No
274	M	61	Tongue	pT2N0M0	S+Postop RT	64	Alive	No
305	M	58	Tongue	pT2N1M0	S+postop CRT	50	Alive	No
306	M	33	Tongue	pT3N3bM0	S+Postop CRT	48	Dead	Yes
307	M	37	Tongue	cT2N0M0	S+postop RT	21	Dead	Yes

312	M	32	Tongue	cT2N0M0	S+Postop RT	43	Alive	No
315	M	60	Tongue	pT3N1M0	S+Postop CRT	41	Alive	No
319	M	78	Tongue	cT3N2cM0	RT	3	Dead	No
320	M	66	Tongue	cT1N0M0	S	25	Dead	Yes
321	F	76	Tongue	pT1N0M0	S	35	Alive	No

F=female. M=male. S=surgery. Postop RT=postoperative radiotherapy (adjuvant). CT=Chemotherapy. RT=Radiotherapy. Preop RT=preoperative radiotherapy (neoadjuvant). Postop CRT=postoperative chemoradiotherapy ((adjuvant). FOM = floor of the mouth. Gingiva = upper and lower gum.

Table S2. Olink Explore 3072 collagen assays and corresponding epitope targets.

Uniprot ID	Gene symbol	Protein Name	Antibody target
**P02452**	COL1A1	Collagen alpha-1(I) chain	N- & C-terminal pro-peptide
**P02458**	COL2A1	Collagen alpha-1(II) chain	Mature collagen epitopes
**P02461**	COL3A1	Collagen alpha-1(III) chain	C-terminal pro-peptide
**P02462**	COL4A1	Collagen alpha-1(IV) chain	Mature collagen epitopes
**P53420**	COL4A4	Collagen alpha-4(IV) chain	Mature collagen epitopes
**P20908**	COL5A1	Collagen alpha-1(V) chain	C-terminal propeptide
**P12111**	COL6A3	Collagen alpha-3(VI) chain	Mature collagen epitopes
**P20849**	COL9A1	Collagen alpha-1(IX) chain	Mature collagen epitopes
**Q14055**	COL9A2	Collagen alpha-2(IX) chain	Mature collagen epitopes
**P39059**	COL15A1	Collagen alpha-1(XV) chain	Mature collagen epitopes
**P39060**	COL18A1	Collagen alpha-1(XVIII) chain	Mature collagen epitopes
**Q17RW2**	COL24A1	Collagen alpha-1(XXIV) chain	Mature collagen epitopes
**Q2UY09**	COL28A1	Collagen alpha-1(XXVIII) chain	Mature collagen epitopes

Table S3. Clinicopathological parameters of participants enrolled in the microarray analysis.

ID	Sample	Age (years)	Sex	TNM
p11	NTCT & Tumor	77	Female	T2N1M0
p14	Tumor	64	Male	T1N0M0
p24	Tumor	64	Female	T2N0M0
p29	Tumor	62	Male	T2N0M0
p35	NTCT & Tumor	71	Male	T1N0M0
p40	NTCT	80	Female	T4N2bM0
p42	NTCT	68	Female	T2N0M0
p49	NTCT & Tumor	19	Female	T4N0M0
p51	NTCT & Tumor	64	Female	T1N0M0
p56	NTCT & Tumor	63	Female	T2N0M0
p58	NTCT & Tumor	78	Male	T2N0M0
p59	NTCT & Tumor	24	Female	T2N0M0
p61	NTCT & Tumor	52	Female	T4N2cM0
p65	NTCT & Tumor	74	Male	T2N0M0
p68	Tumor	40	Female	T2N2bM0
p70	Tumor	61	Male	T1N0M0
p73	NTCT & Tumor	68	Female	T2N0M0
p76	NTCT & Tumor	69	Male	T4aN0M0
p79	NTCT & Tumor	81	Female	T2N0M0
p82	Tumor	80	Male	T4aN0M0
p83	Tumor	58	Male	T4aN0M0
p85	NTCT & Tumor	60	Male	T1N0M0
p92	Tumor	87	Female	T2N0M0
p98	NTCT & Tumor	31	Male	T2N0M0
p105	NTCT & Tumor	63	Male	T1N0M0
p111	NTCT & Tumor	31	Female	T1N0M0
p119	NTCT & Tumor	66	Male	T2N0M0
p124	NTCT & Tumor	54	Male	T4aN2bM0
p131	NTCT & Tumor	74	Female	T2N0M0
p137	NTCT & Tumor	71	Female	T2N0M0
p138	NTCT & Tumor	50	Male	T2N1M0
H1	Healthy control	32	Female	H1
H2	Healthy control	49	Female	H2
H3	Healthy control	25	Female	H3
H4	Healthy control	30	Male	H4
H5	Healthy control	27	Male	H5
H6	Healthy control	42	Female	H6
H7	Healthy control	32	Female	H7
H8	Healthy control	41	Female	H8
H9	Healthy control	35	Female	H9
H10	Healthy control	57	Male	H10
H11	Healthy control	45	Male	H11
H12	Healthy control	37	Male	H12

H13	Healthy control	48	Female	H13
H14	Healthy control	59	Female	H14

Table S4. Associations between plasma collagen levels and clinical parameters.

Collagen type	Age		Sex	Tumor size	Node status
	rho	*p*-value	*p*-value	*p*-value	*p*-value
COL1A1	-0.434	< .001	0.398	0.1	0.64
COL2A1	-0.381	< .001	0.269	0.899	0.495
COL3A1	-0.279	0.016	0.671	0.921	0.698
COL4A1	0.079	0.502	0.893	0.944	0.602
COL5A1	-0.319	0.006	0.876	0.453	0.414
COL6A3	0.235	0.044	0.91	0.543	0.64
COL9A1	-0.32	0.005	0.299	0.357	0.547
COL15A1	-0.067	0.571	0.209	0.47	0.289
COL18A1	0.538	< .001	0.067	0.127	0.429
COL24A1	-0.126	0.284	0.953	0.002	0.779
COL28A1	0.171	0.146	0.515	0.661	0.211

rho: Spearman correlation coefficient

Table S5. Differentially abundant proteins between COL3A1-low and COL3A1-high patients.

Protein	P.Value	adj.P.Val	log2 fold change
COL3A1	3.90E-08	1.15E-04	0.471
SNU13	6.78E-04	9.68E-01	-0.863
CBLIF	1.21E-03	9.68E-01	1.029
CEACAM8	1.80E-03	9.68E-01	-0.801
PINLYP	1.81E-03	9.68E-01	0.797
ATXN2L	2.19E-03	9.68E-01	-0.386
LIPF	2.74E-03	9.68E-01	0.711
ASAH2	2.80E-03	9.68E-01	0.460
EP300	3.61E-03	9.68E-01	-0.280
CPQ	4.13E-03	9.68E-01	0.355
MAPK13	5.32E-03	9.68E-01	-0.316
RUVBL1	8.38E-03	9.68E-01	-0.838
COL1A1	8.41E-03	9.68E-01	0.428
USP47	8.55E-03	9.68E-01	-0.531
THBS2	8.61E-03	9.68E-01	0.542
DDC	8.77E-03	9.68E-01	0.614
ENO3	9.31E-03	9.68E-01	0.789
CXCL17	9.99E-03	9.68E-01	0.498
PGLYRP1	1.02E-02	9.68E-01	-0.366
CEBPA	1.02E-02	9.68E-01	-0.252
CLEC3B	1.04E-02	9.68E-01	0.186
FLI1	1.16E-02	9.68E-01	-0.488
PADI2	1.20E-02	9.68E-01	-0.647
SIRT1	1.31E-02	9.68E-01	-0.429
RNASE3	1.37E-02	9.68E-01	-1.475
MPO	1.40E-02	9.68E-01	-0.657
CXCL6	1.41E-02	9.68E-01	-0.555
CCN5	1.42E-02	9.68E-01	-0.300
LAMP1	1.49E-02	9.68E-01	0.238
HDGF	1.55E-02	9.68E-01	-0.783
CXCL1	1.55E-02	9.68E-01	-0.661
GPKOW	1.64E-02	9.68E-01	-0.304
LILRA6	1.64E-02	9.68E-01	-0.596
LCN2	1.69E-02	9.68E-01	-0.364
MSLNL	1.72E-02	9.68E-01	-0.218

AZU1	1.92E-02	9.68E-01	-0.926
IGHMBP2	1.95E-02	9.68E-01	-0.514
MKI67	1.96E-02	9.68E-01	-0.531
PAMR1	1.97E-02	9.68E-01	0.267
SSB	2.01E-02	9.68E-01	-0.366
NECAP2	2.06E-02	9.68E-01	-0.489
PGA4	2.08E-02	9.68E-01	0.671
SERPINA7	2.12E-02	9.68E-01	0.161
CTSH	2.12E-02	9.68E-01	0.529
CST1	2.16E-02	9.68E-01	-1.205
ZCCHC8	2.18E-02	9.68E-01	-0.553
TOP1	2.21E-02	9.68E-01	0.969
RETN	2.22E-02	9.68E-01	-0.335
OSM	2.24E-02	9.68E-01	-0.858
SERPINB8	2.29E-02	9.68E-01	-0.611
HEPH	2.30E-02	9.68E-01	0.210
BTD	2.34E-02	9.68E-01	0.177
DEFA1	2.60E-02	9.68E-01	-0.425
MNAT1	2.63E-02	9.68E-01	-0.382
EPO	2.65E-02	9.68E-01	-0.484
MMP8	2.66E-02	9.68E-01	-0.749
ARID3A	2.68E-02	9.68E-01	-0.392
CDCP1	2.75E-02	9.68E-01	0.437
RFC4	2.76E-02	9.68E-01	-0.142
CDK1	2.82E-02	9.68E-01	-0.129
LPCAT2	2.86E-02	9.68E-01	-0.690
JUN	2.87E-02	9.68E-01	-0.236
FCGR2A	2.87E-02	9.68E-01	0.399
BAMBI	2.92E-02	9.68E-01	-0.100
COL5A1	2.96E-02	9.68E-01	0.349
LILRB2	3.01E-02	9.68E-01	0.247
TPR	3.02E-02	9.68E-01	-0.786
ENPP7	3.03E-02	9.68E-01	0.704
GOLM2	3.09E-02	9.68E-01	-0.182
OLR1	3.19E-02	9.68E-01	-0.727
DDA1	3.21E-02	9.68E-01	-0.621
S100P	3.21E-02	9.68E-01	-0.418

PRKRA	3.21E-02	9.68E-01	-0.576
PRND	3.22E-02	9.68E-01	0.518
CD207	3.22E-02	9.68E-01	0.320
DGCR6	3.28E-02	9.68E-01	-0.338
CDH6	3.34E-02	9.68E-01	0.302
PQBP1	3.42E-02	9.68E-01	-0.681
GLYR1	3.44E-02	9.68E-01	-0.605
CWC15	3.55E-02	9.68E-01	-0.659
APOBR	3.55E-02	9.68E-01	0.318
PLB1	3.59E-02	9.68E-01	0.463
NBN	3.61E-02	9.68E-01	-0.674
MMP9	3.65E-02	9.68E-01	-0.562
LBR	3.70E-02	9.68E-01	-0.772
AKR7L	3.73E-02	9.68E-01	0.572
PM20D1	3.76E-02	9.68E-01	1.249
SRP14	3.82E-02	9.68E-01	-0.257
VNN2	3.87E-02	9.68E-01	-0.339
FAS	3.90E-02	9.68E-01	0.215
FLT3LG	3.99E-02	9.68E-01	0.333
CAMLG	4.01E-02	9.68E-01	-0.445
TIA1	4.10E-02	9.68E-01	-0.271
SMPDL3B	4.12E-02	9.68E-01	-0.301
PCDHB15	4.14E-02	9.68E-01	0.327
C1QTNF9	4.17E-02	9.68E-01	0.301
CDON	4.23E-02	9.68E-01	0.227
SPINK6	4.29E-02	9.68E-01	-0.374
NFIC	4.38E-02	9.68E-01	-0.257
FUS	4.41E-02	9.68E-01	-0.385
PRTN3	4.49E-02	9.68E-01	-0.574
VIM	4.51E-02	9.68E-01	-0.520
MFAP4	4.51E-02	9.68E-01	0.213
LYPD3	4.61E-02	9.68E-01	0.222
NPTX1	4.65E-02	9.68E-01	-0.270
RNASEH2A	4.71E-02	9.68E-01	-0.367
ADAMTS15	4.77E-02	9.68E-01	0.295
ZNRF4	4.83E-02	9.68E-01	-0.311
ARID4B	4.94E-02	9.68E-01	-0.246

FMNL1	4.96E-02	9.68E-01	-0.785
ENG	4.97E-02	9.68E-01	0.116
TSPAN15	4.99E-02	9.68E-01	-0.284
